# Molecular Characterization and Functional Localization of a Novel SUMOylation Gene in *Oryza sativa*

**DOI:** 10.3390/biology11010053

**Published:** 2021-12-31

**Authors:** Eid I. Ibrahim, Kotb A. Attia, Abdelhalim I. Ghazy, Kimiko Itoh, Fahad N. Almajhdi, Abdullah A. Al-Doss

**Affiliations:** 1 Biotechnology Lab., Plant Production Department, College of Food and Agriculture Sciences, King Saud University, P.O. Box 2460, Riyadh 11451, Saudi Arabia; aghazy@ksu.edu.sa (A.I.G.); aaldoss@ksu.edu.sa (A.A.A.-D.); 2 Center of Excellence in Biotechnology Research, King Saud University, P.O. Box 2455-11451, Riyadh 11451, Saudi Arabia; 3 Rice Biotechnology Lab., Rice Research Department, Field Crops Research Institute, ARC, Sakha, Kafr, EL-Sheikh 33717, Egypt; 4 Institute of Science and Technology, Niigata University, Niigata 950-2181, Japan; kimi.itoh@agr.niigata-u-ac.jp; 5 Department of Botany and Microbiology, College of Science, King Saud University, P.O. Box 2455, Riyadh 11451, Saudi Arabia; majhdi@ksu.edu.sa

**Keywords:** rice, SUMO genes, bioinformatic analysis, gene expression, cellular localization

## Abstract

**Simple Summary:**

The small ubiquitin-related modifier genes regulate the function of the cellular proteins, which are associated with cell stress-tolerance. Identification and understanding the functional localization of these genes are very important to mitigate the stresses. In this study, we identified a novel small ubiquitin-related modifier gene and studied its functional localization in the cell. This new finding will be very valuable in increasing our understanding of the mechanism of stress-tolerance.

**Abstract:**

Small ubiquitin-related modifier (SUMO) regulates the cellular function of diverse proteins through post-translational modifications. The current study defined a new homolog of SUMO genes in the rice genome and named it *OsSUMO7*. Putative protein analysis of *OsSUMO7* detected SUMOylation features, including di-glycine (GG) and consensus motifs (ΨKXE/D) for the SUMOylation site. Phylogenetic analysis demonstrated the high homology of *OsSUMO7* with identified rice SUMO genes, which indicates that the *OsSUMO7* gene is an evolutionarily conserved SUMO member. RT-PCR analysis revealed that *OsSUMO7* was constitutively expressed in all plant organs. Bioinformatic analysis defined the physicochemical properties and structural model prediction of OsSUMO7 proteins. A red fluorescent protein (DsRed), fused with the OsSUMO7 protein, was expressed and localized mainly in the nucleus and formed nuclear subdomain structures. The fusion proteins of SUMO-conjugating enzymes with the OsSUMO7 protein were co-expressed and co-localized in the nucleus and formed nuclear subdomains. This indicated that the OsSUMO7 precursor is processed, activated, and transported to the nucleus through the SUMOylation system of the plant cell.

## 1. Introduction

Post-translational modifications (PTMs) of proteins play an essential role in a majority of various cellular processes [1,2,3]. PTMs of proteins occur through various mechanisms such as acetylation, phosphorylation, glycosylation, ubiquitination (Ub), and SUMOylation [4,5]. The small ubiquitin-related modifier (SUMO) is an essential regulator of different biological processes [3,6]. SUMO, a low-molecular-weight protein about 100 amino acids in length, is a form of a ubiquitin-like protein (Ubl) in structure but is different in function [7]. The inconsistency of this functional mechanism is because ubiquitination is involved in proteasomal degradation while SUMOylation suppresses this degradation to change the target protein’s localization [8,9,10]. The SUMO protein is present in all eukaryotic cells and has emerged as an influential mechanism for target protein management [1,11]. SUMO has a consolidated center sequence as well as N- and C-variable terminal sequences. It is only approximately 20 percent similar to Ub, but comparable to Ub in that it has a three-dimensional (3D) structure [12]. The SUMO system contains several protein isoforms which participate in SUMOylation reactions [4]. SUMOylation is a mechanism that occurs in the nucleus and cytoplasm via a cascade of parallel enzymatic reactions, including activation enzymes (E1-SUMO), conjugation enzymes (E2-SUMO), and ligation enzymes (E3-SUMO) [13,14]. It often occurs in lysine (K) inside the SUMO consensus sequence ΨKXE/D (ψ, a hydrophobic residue; K, lysine; X, any amino acid; and E/D, glutamic, or aspartic) [11,15,16]. SUMOylation plays a main role in several important intracellular processes, such as the activity and stability of enzymes, DNA repair, and cell cycle regulation [3,17,18]. The SUMO system affects plant production through the accumulation of SUMO conjugates under abiotic stress [3,19,20,21]. In *Arabidopsis*, the abundance of SUMO conjugates and increases in response to salinity, heat, and brassinosteroid signaling [22,23,24,25]. The E3 ligase *SIZ1* (SAP and miz1) regulates plant development and high-temperature stress in *Arabidopsis* as well as improved water deficit stress tolerance in tobacco [26,27]. Esd4 (early in short days 4) is a form of a SUMO-specific protease that controls flowering time and promotes the SUMO precursor in *Arabidopsis* [28]. It has also been found that the SUMO protease *OTS1* (overly tolerant to salt 1) is a positive regulator for rice seed germination and root system growth under salt stress [29]. The SUMOylation of OsSIZ1 regulated the response to phosphate and nitrogen, as well as the Pi-dependent response in rice [30]. *OsMMS21* is the SUMO-ligase-regulated auxin response and development in rice [31]. Overexpression of the *ZmSCE1e* (*Zea mays* SUMO conjugation enzyme 1) maize gene increased the level of SUMO conjugation and amplified the tolerance to drought and salinity of transgenic tobacco [32]. Eight SUMO genes were reported in the *Arabidopsis thaliana* genome [33], and its SUMO proteins were classified into two groups [34,35]. The first group was mono-SUMOylation (mono-SUMO), which contained amino acid sequences without SUMO acceptor sites, whereas the second group was poly-SUMOylation (poly-SUMO), which had amino acid sequences with one or more SUMO acceptor sites [36]. In the rice genome, six SUMO genes have been reported so far, named *OsSUMO1–6* [16,18,37]. In the current investigation, we defined and molecularly characterized a new rice SUMO family member. Additionally, we analyzed the expression patterns and cellular localization of its SUMO protein in plant cells.

## 2. Materials and Methods

### 2.1. Plant Material and Candidate Gene Amplification

Seedlings of *Oryza sativa* L., cv Super-300 (Egyptian Japonica variety) were germinated and grown in a greenhouse under conditions of 14.5 L/9.5 D day length as well as 30 °C day/25 °C night thermo-conditions at an experimental farm of the Rice Research & Training Center (RRTC), Field Crops Research Institute, Sakha, Egypt. Molecular work was achieved at the Biotechnology Lab of the Plant Production Department, Food, and Agriculture College, King Saud University, Riyadh, Saudi Arabia. The extraction of total RNA from young rice leaves was performed using a plant total RNA extract kit (Magen) and then used for first-strand cDNA synthesis with SuperScript II (Invitrogen, currently Life-Technologies Co., San Francisco, CA, USA). The coding region of *OsSUMO7* was amplified using specific primers, 5′ TGCCTTCCATCGTTGTGTTG ′3 (forward) and 5′ CCATATCAACTTCAGCAGATTCC ′3 (reverse), which were designed from genomic DNA of the SUMO region of a Japonica rice genome database. The PCR amplification reaction was completed using 12.5 μL of 1 × of GoTaq^®^ Green Master Mix (Promega Corporation, Madison, WI, USA), 2-μL mix primers, 8.5 μL of double-distilled water, and 2 μL of DNA. The thermo-cycling conditions were 95 °C for 5 min for initial denaturation, followed by 35 cycles at 95 °C for 30 s; 57 °C for 45 s; 72 °C for 1 min; and 72 °C for 10 min for extra elongation. Ten micro-liters from the PCR product was used for 2% agarose gel electrophoresis to check the target band of the candidate gene.

### 2.2. RNA Extraction and Reverse-Transcription PCR

To investigate the transcription levels of *OsSUMO7*, different organs of the Japonica-type rice were collected and grinded with liquid nitrogen for RNA extraction using a plant total RNA extract kit (Magen). RT-PCR was performed using KOD Dash DNA polymerase (Toyobo) and an ABI 2720 thermal cycler (Life Technologies, Carlsbad, CA, USA). The transcript of the new SUMO gene was amplified using a pair of gene-specific primers: 5′ GGGTTGATTGATGGTGGTGG ′3 (forward) and 5′ GTGAAACGGAGGAAGTAGCTC ′3 (reverse). Rice *Actin1* was used as an internal control.

### 2.3. Sequencing and Bioinformatic Analysis of OsSUMO7

Sequencing of the candidate region: The expected PCR band was purified using a Gel Extraction Kit (Qiagen Inc., Chatsworth, California, CA, USA) and sequenced using an automated DNA sequencer (Applied Biosystems Inc., Foster City, California, CA, USA). Six rice SUMO genes have been reported [18,38]; we obtained their sequences from the Rice Annotation Project (http://rapdb.dna.affrc.go.jp/, accessed on 6 October 2021). Known protein/DNA sequences were used as search queries to BLAST against rice sequence databases to determine if the rice genome encodes additional SUMO proteins. Arabidopsis *AtSUMO* sequences were obtained from TAIR (http://www.arabidopsis.org, accessed on 6 October 2021), while human and yeast SUMO sequences were obtained from the NCBI database. Sequences were aligned using ClustalW and then searched for conserved domains using the Pfam database [39,40]. Protein sequences alignment was performed using ClustalX software (http://www.clustal.org/, accessed on 6 October 2021). Phylogenetic tree analysis was carried out using the neighbor-joining method, PHYLIP version 3.69 (http://evolution.genetics.washington.edu/phylip.html, accessed on 6 October 2021), with bootstrap values from 1000 neighbor-joining bootstrap replicates. The candidate gene-coding region was investigated to define the SUMO features.

### 2.4. In Silico Characterization of the OsSUMO7 Protein

SUMOplot^TM^ prediction: the SUMO binding sites in SUMO genes were identified using computer SUMOplot™ software. Prediction of the structure of OsSUMO7: the prediction of homology, secondary structure, and three-dimensional (3D) structure were carried out using the SWISS-MODEL, I-TASSER server (https://zhanglab.ccmb.med.umich.edu/I-TASSER/, accessed on 6 October 2021) [41]. Physicochemical properties of the SUMO proteins were determined using the ExPASy ProtParam server tool [42]. Primary structure, such as molecular weight (MW), the composition of amino acids, atomic composition, and the theoretical isoelectric point (T-pi), were calculated. In addition, the instability index (Ii) was calculated by Guruprasad [43], showing that there are certain dipeptides, the occurrence of which is significantly different in the unstable proteins compared with those in the stable ones. A protein with an instability index value of less than 40 is stable, while those with more than 40 are unstable. The aliphatic index (Ai) of a protein is obtained by using the formula described by Ikai [44]. The grand average of hydropathy (GRAVY) is calculated by the methodology reported by Kyte [45].

### 2.5. Construction of Expression Vectors Harboring Rice SUMO Components

The original expression vector *pUC119* [46] has been used to construct the expression vectors harboring OsSUMO components according to the methodology described by Ikarashi [37]. The constructed vectors expressed red or green fluorescent protein makers (DsRed or GFP), driven by the cauliflower mosaic virus 35S (CaMV35S) promoter (Figure S1). All the corresponding primers that were used in the study are listed in Table S1. The target genes were inserted into pDsRed using an In-Fusion HD Cloning Kit (Takara Bio, Kusatsu, Japan), and the reconstructed vectors were designated as follows. The vector *pDsRed:SUMO7* was constructed to investigate the expression and localization of the new OsSUMO7 protein. A series deletion mutant vector, *pDsRed:SUMO7*Δ*GG*, was constructed to study the role of the GG motif on the cellular localization of OsSUMO7 proteins. The SUMO conjugation enzyme (SCE1a) was cloned into a *pGFP:SCE1a* vector to study its co-expression and co-localization with OsSUMO7 proteins. Cell organellar markers were fused with GFP and constructed vectors, such as *pPTS2:GFP* (peroxisomal marker), *pmt:GFP* (mitochondrial marker), *pWxTP:GFP* (plastidial marker), and *pGFP:SYP31* (cis-Golgi marker) (Figure S1). These vectors were co-transfected with *DsRed:SUMO7*Δ*GG* in onion cells to study whether the OsSUMO7 protein is localized in any other organelles in the cell.

### 2.6. Expression Analysis of SUMO Proteins

Constructed vectors harboring SUMO genes were transformed into onion epidermal cells using particle bombardment (Biolistic^®^ PDS-1000/He, BioRad). Bombarded cells were kept in the dark for 24 h at room temperature. The expressed proteins were observed using the confocal laser scanning microscopy (FV300-BX61, Olympus), according to the methods reported by Ikarashi and Kitajima, respectively [37,47].

## 3. Results

The novel *SUMO* gene of rice was identified using the known SUMO system pathway components from Arabidopsis. The Arabidopsis SUMO protein/gene sequences were downloaded from the website genome database (https://www.arabidopsis.org/, accessed on 6 October 2021). The basic local alignment search tool for amino acids (BLASTP) and the basic local alignment search tool for nucleotides (TBLASTN) analyses were performed with the rice genome database (https://rapdb.dna.affrc.go.jp/, accessed on 6 October 2021) using the identified Arabidopsis *SUMO* genes. From the outcomes, the top identity sequences were chosen. Reciprocal BLASTP/TBLASTN analysis were performed using National Center for Biotechnology Information (NCBI) to ensure that the subject is most closely matched to the SUMO pathway query. All previously identified *SUMO* genes in rice were excluded. PCR primers were designed for the highest similarity sequence with the rice genome crops.

### 3.1. Molecular Characterization of the OsSUMO7 Gene

**Figure 1:**
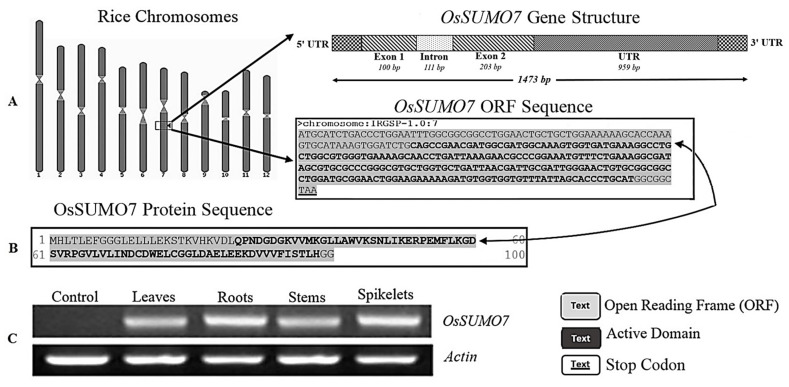
(**A**) Rice chromosomes and the genomic sequence of the *OsSUMO7* gene. (**B**) OsSUMO7 protein sequence. (**C**) Transcripts level of OsSUMO7 using RT-PCR.

**Figure 2:**
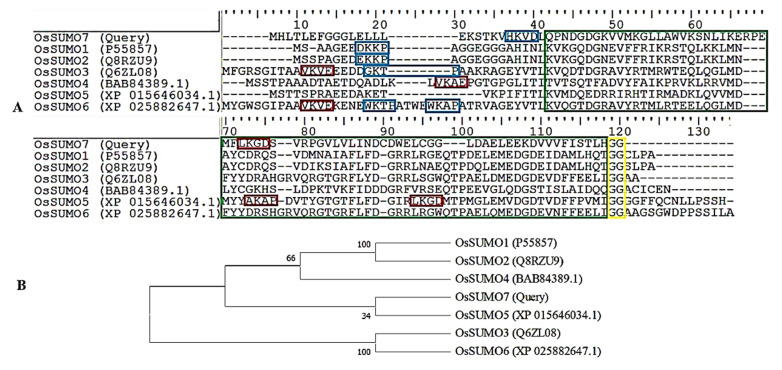
(**A**) Alignment of *SUMO* genes in rice. Di-glycine motifs at the end of the mature SUMO proteins are indicated by the yellow box. Potential SUMOylation sites (ΨKXE/D) are boxed in red for a high probability and with blue for a low probability. A green box refers to the conserved ubiquitin-like domain. (**B**) Phylogenetic tree analysis of the OsSUMO family.

**Figure 3:**
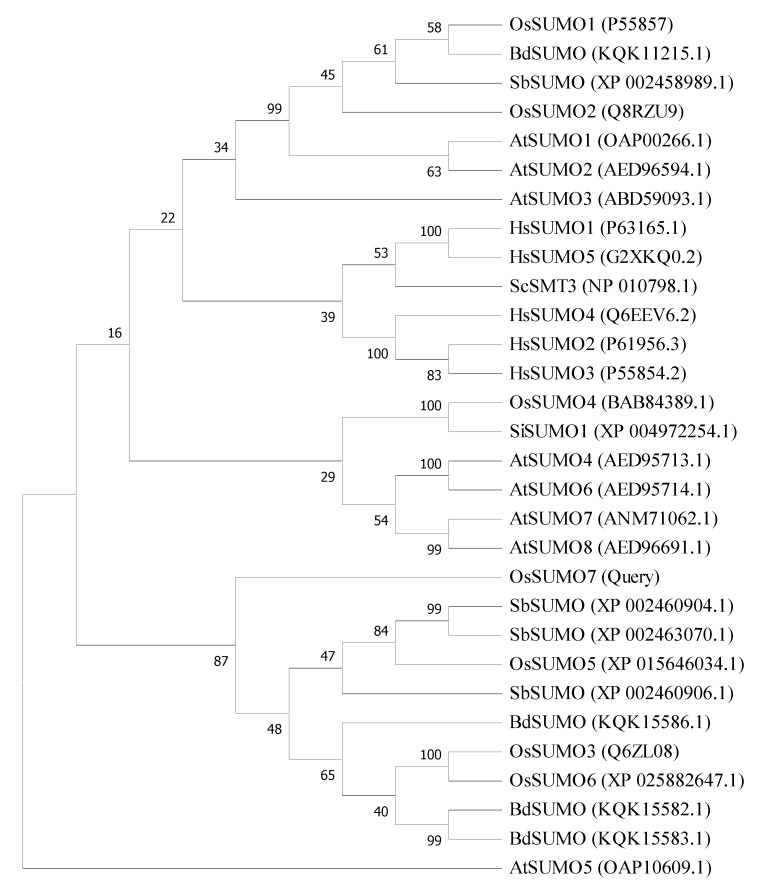
Phylogenetic tree analysis of *SUMO* genes. Multiple sequence alignment result of *SUMOs* from ClustalW. Os refers to *Oryza sativa*, At refers to *Arabidopsis thaliana*, Hs refers to *Homo sapiens*, Bd refers to *Brachypodium distachyon*, Si refers to *Setaria italic*, Sb refers to *Sorghum bicolor*, and Sc refers to *Saccharomyces cerevisiae*.

Using PCR, we amplified the coding region of a new SUMO family’s genes from the rice genome using specific primers (Table S1), which were designed based on genomic DNA of the SUMO region from a *Japonica* rice genome database (Figure 1A). The sequencing analysis determined that the 303 bps region coded the complete protein of 100 amino acids length (Figure 1B). Transcriptional levels of *OsSUMO7* were investigated using RT-PCR in different rice organs, such as leaves, roots, stem, and spikelets. The data revealed that the gene was expressed in the tested rice organs (Figure 1C), indicating the constitutive expression of the gene. A BLAST analysis based on the protein sequence was performed in the rice database and showed that the *OsSUMO7* gene predicted a protein containing 100 amino acid residues. These amino acid sequences have a C-terminal diglycine (GG) motif as the processing site by ULP; the consensus motifs (ΨKXE/D) of the SUMOylation site showed a high similarity to other SUMO members (Figure 2A). These data indicated that the *OsSUMO7* gene has the features of SUMO members. A phylogenetic tree was generated based on the predicted SUMO protein sequences from different species, including *Oryza sativa* (Os), *Arabidopsis thaliana* (At), *Homo sapiens* (Hs), *Brachypodium distachyon* (Bd), *Setaria italic* (Si), *Sorghum bicolor* (Sb), and *Saccharomyces cerevisiae* (Sc). The OsSUMO7 protein shared a higher homology with monocotyledons, such as *Brachypodium distachyon* (Bd), *Oryza sativa* (Os), and *Sorghum bicolor* (Sc) (Figure 3). The similarity of protein sequences and conserved domains among OsSUMOs of different species demonstrated that OsSUMO7 is an evolutionarily conserved SUMO family member (Figure 2B).

### 3.2. Characterization of the OsSUMO7 Protein:

A bioinformatic analysis was performed to characterize the OsSUMO7 protein, comparing it to other OsSUMO and AtSUMO members. The results indicated that the *OsSUMO7* gene located on chromosome seven was similar to *OsSUMO3,5,6* genes and coded full-length active proteins with 100 amino acids, similar to *OsSUMO1* (Table 1). Moreover, the OsSUMO7 protein contains two motifs for SUMOylation sites, one with a high probability at the K58 position and another with a low probability at the K23 position, similar to *OsSUMO6*; however, *OsSUMO6* has two low-probability motifs at the K20 and K28 positions (Table 1).

#### 3.2.1. Physicochemical Properties Analyses

**Figure 4:**
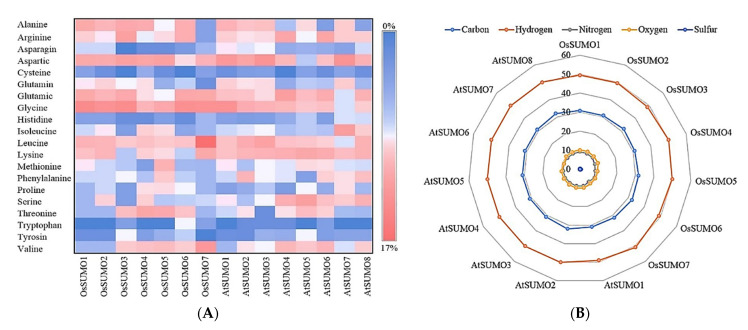
(**A**) Heatmap presentation of OsSUMOs’ and AtSUMOs’ composition of amino acid by percentage. The common amino acid present in all SUMOs is shown. (**B**) Radar graph for atomic composition percentage (number of each atom by number of total atoms) of OsSUMOs and AtSUMOs.

Physicochemical properties analyses of SUMO proteins, performed using ProtParam, are listed in Table 2. The results recorded that the MW of the OsSUMO7 protein is 10.99 KDa, similar to the OsSUMO1, AtSUMO1, and AtSUMO7 proteins. The OsSUMO7 protein recorded a T-Pi value of 4.92, which is almost equal to OsSUMO1 and AtSUMO1. In addition, the OsSUMO7 protein revealed a TNNCR (Asp + Glu) value (17) similar to OsSUMO2 and OsSUMO4, while it showed the lowest TNPCR (Arg + Lys) value (11) among all the SUMO proteins (Table 2). The instability index (Ii) value was less than 40 (30.35) for OsSUMO7, indicating the stability of its protein, which was similar to the proteins of OsSUMO4, OsSUMO5, AtSUMO2, and AtSUMO7; the other SUMO proteins were unstable. At the same time, the OsSUMO7 protein recorded the highest value of Ai (119) and GRAVY (0.031) among all the tested SUMO proteins (Table 2). The amino acid composition analysis demonstrated that the OsSUMO7 protein sequence included all 20 amino acids, except tyrosine (Y) (Figure 4A, Table S2). In addition, it showed the lowest percentage of Ala (A, 2%), Arg (R, 2%), and Gln (Q, 1%) amino acids among the tested SUMO proteins, whereas the OsSUMO7 protein recorded the highest percentage of Leu (L, 17%) and Val (V, 11%) amino acids among the other SUMO proteins that were tested. Concurrently, the OsSUMO7 protein revealed high percentages of Gly (G, 12%), Lys (K, 9%), Glu (E, 9%), and Asp (D, 8%) (Figure 4A, Table S3). The atomic composition (AC) of the amino acid included carbon (C), hydrogen (H), nitrogen (N), oxygen (O), and sulfur (S). The results showed that the OsSUMO6 recorded the highest value (2034) of TNA, while the OsSUMO7 protein recorded a value of 1565 of TNA (Table S2). Radar graph analysis for atomic composition demonstrated that there was no significant difference in atomic composition percentage among the tested SUMO proteins (Figure 4B).

#### 3.2.2. Structure–Model Prediction of the OsSUMO7 Protein

**Figure 5:**
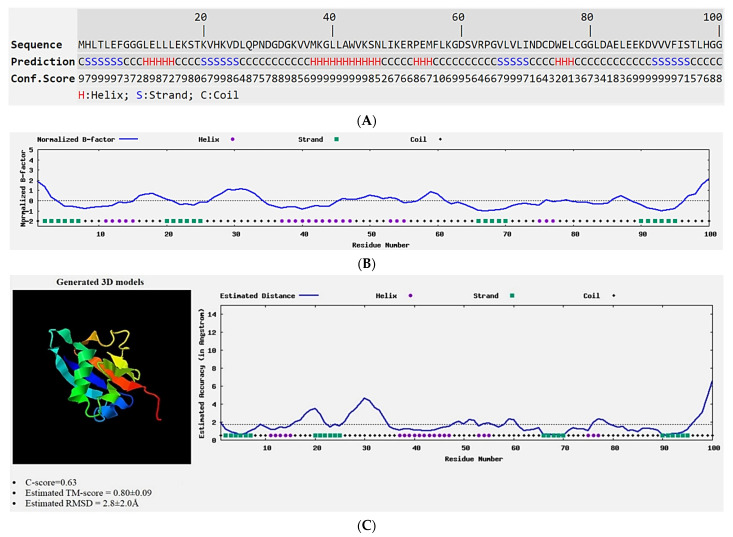
(**A**) Putative OsSUMO7 protein prediction of secondary structure by PSSpred. (**B**) The predicted normalized B-factor of OsSUMO7 by ResQ. (**C**) The predicted 3D model, as well as the estimated global and local accuracy of the OsSUMO7, calculated by the I-TASSER server.

The analysis of the secondary structure was determined based on sequence-based prediction using PSSpred. The data showed that the sequence’s structure identity (helix, strand, or coil) is determined by the sum of the confidence scores. The structure revealed four strands intervened by eight coils and four helixes (Figure 5A). The confidence scores showed relatively high values, which are indicative of high-confidence predictions. Likewise, the predicted normalized B-factor performed using the ResQ method revealed the similarity with the secondary structure of the OsSUMO7 sequence model, which showed four strands intervened by eight coils and four helixes (Figure 5B). The negative values indicated the stability of the OsSUMO7 structure. The SPIKER program was used to select for a 3D model produced by I-TASSER simulations based on the pairwise structure similarity. The final predicted 3D model for OsSUMO7 observed an estimated TM-score value of 0.80 ± 0.09, a C-score of 0.63, and a calculated RMSD of 2.8 ± 2.0 A° (Figure 5C).

### 3.3. Expression and Subcellular Localization of the OsSUMO7 Protein

**Figure 6:**
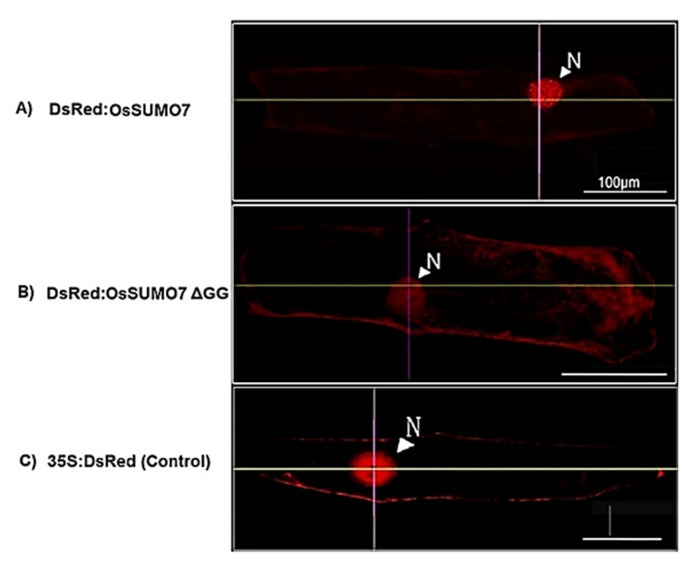
(**A**) Localization of DsRed:OsSUMO7 in onion cells. (**B**) Localization of DsRed:OsSUMO7 with GG deletion in onion cells. (**C**) 35S:DsRed (control).

**Figure 7:**
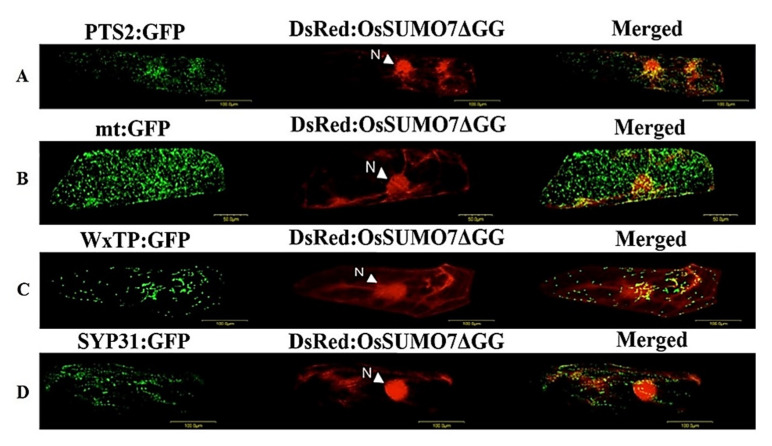
(**A**) Co-expression of DsRed:OsSUMO7ΔGGs and PTS2:GFP in onion cells. PTS2 peroxisomal marker. (**B**) Co-expression of DsRed:OsSUMO7ΔGGs and mt:GFP in onion cells. mt; mitochondrial marker. (**C**) Co-expression of DsRed:OsSUMO7ΔGGs and WxTP:GFP in onion cells. WxTP, plastidial marker. (**D**) Co-expression of DsRed:OsSUMO7ΔGGs and SYP31:GFP in onion cells. SYP31, cis-Golgi marker.

The DsRed:SUMO7 fusion proteins were mainly localized in the nucleus and form nuclear subdomains (Figure 6A). Moreover, *DsRed:SUMO7*Δ*GG* fusion proteins were detected in both the cytoplasm and nucleus. However, subnuclear structures disappeared (Figure 6B). To study whether the SUMO proteins are localized in any other organelles in the cell, various constructed vectors expressing some organellar markers, including *pPTS2:GFP* (peroxisomal marker), *pmt:GFP* (mitochondrial marker), *pWxTP:GFP* (plastidial marker), and *pGFP:SYP31* (cis-Golgi marker), were constructed, and transfection was performed with pDsRed:OsSUMO7 in onion epidermal cells. The results showed that the OsSUMO7 proteins did not localize at any of these organelles (Figure 7).

### 3.4. Expression and Co-Localization of OsSUMO7 with GFP:SCE1a

**Figure 8:**
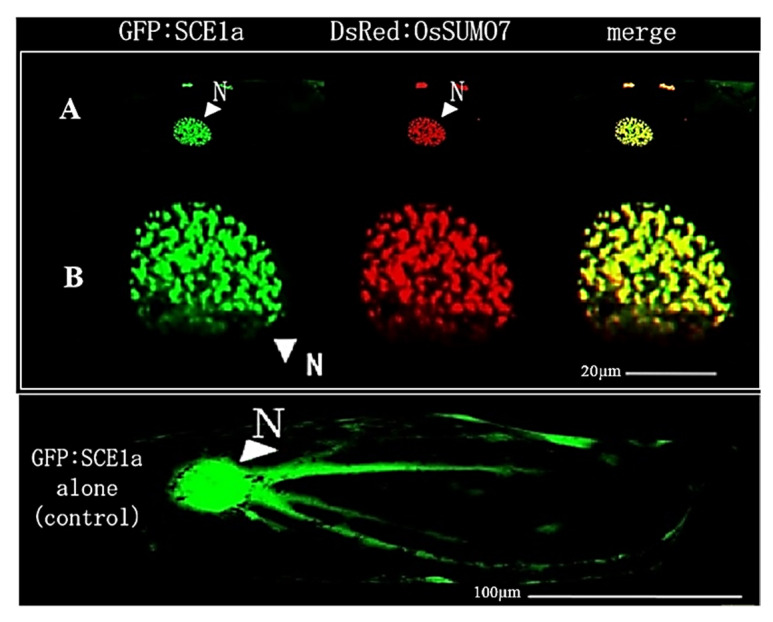
(**A**) Co-expression and co-localization of DsRed:OsSUMO7 with GFP:SCE1a in the nucleus of onion cells. (**B**) Co-expression and co-localization of DsRed:OsSUMO7 with GFP:SCE1a in the nucleus with high magnification.

The fusion proteins of GFP:SCE1a (SUMO conjugation enzyme) and DsRed:SUMO7, which were co-expressed and co-localized in the nucleus and formed nuclear subdomains, seem to be speckled (Figure 8A). The high-magnification images (Figure 8B) indicated that DsRed:SUMO7 is processed and conjugated within SCE1a:GFP and that both are localized in nuclear foci.

## 4. Discussion

The current investigation defined a new member of SUMO genes in the rice genome and was named *OsSUMO7*. The putative protein sequences of this gene showed to have SUMOylation features, such as di-glycine (GG) motifs at the C-terminal and a consensus motif (ΨKXE/D) at the SUMOylation site (Figure 2A). Many of the SUMO genes were reported in different plant species. Six SUMO genes were identified in the rice genome, named *OsSUMO1~6* [16,18,37]. On the other hand, eight SUMO genes were identified in the Arabidopsis genome, named *AtSUMO1~8* [48]. Most of those SUMO proteins contain consensus motifs at the SUMOylation sites [48,49], except for OsSUMO1/2 genes [18,50]. Among the identified *OsSUMO* genes, the new *OsSUMO7* gene showed high similarity to the *OsSUMO5* gene (Figure 2B). Coincidentally, the OsSUMO7 putative protein shared a higher homology with monocotyledons, such as *Brachypodium distachyon* (Bd), *Oryza sativa* (Os), and *Sorghum bicolor* (Sc) (Figure 3). It was classified in the same cluster as *OsSUMO3/5/6* genes, which might have some important functions. This finding demonstrated that OsSUMO7 is an evolutionarily conserved SUMO member with other homologs of SUMO families in different species. This was consistent with a previous study that reported that the *OsSUMO3/5/6* genes were classified into one group of monocotyledon species [18].

Our data showed that the *OsSUMO7* gene was located on chromosome seven, i.e., the same as the *OsSUMO 3,5,6* genes, while the *OsSUMO1/2/4* genes were located on chromosome one (Table 1). Moreover, a phylogenetic tree classified the OsSUMO proteins into pairs: OsSUMO1/2, OsSUMO7/5, and OsSUMO3/4, while the OsSUMO4 gene was associated with OsSUMO1/2, but not in pairing (Figure 2B). This result suggested that the *OsSUMO1/2/3/5/6/7* genes might be arranged as tandem pairs in the rice genome. Similarly, in the *Arabidopsis* genome, the *AtSUMO2/3/4/6* genes were found to be located on chromosome five in tandem pairs [50,51]. These results indicated that the diversification of rice SUMO genes is similar to those in *Arabidopsis* in tandem duplications.

The transcripts of OsSUMO7 were detected in different rice organs, such as leaves, roots, stem, and spikelets (Figure 1C), which demonstrated the constitutive expression of this gene in all plant organs with no specific tissue. A previous study reported that the transcripts of *OsSUMO1* and *OsSUMO2* functioned in the whole plant body, while the transcripts of *OsSUMO3* were detected only in root and panicle tissues. However, OsSUMO4 transcripts were not detected in any of the rice organs, which suggested that the *OsSUMO4* gene may not be expressed [50]. Conversely, another study reported that all rice SUMO genes were expressed in all plant organs, except for the *OsSUMO4/6* genes which were found to be tissue-specific, functionally expressed in some specific rice organs as opposed to others [18]. This indicated that individual SUMO genes might have diverged functions.

SUMOylation is well known to play a main role in diverse cellular processes, such as stress response, hormone signaling, and development [2,17,29]. Defining the expression levels and cellular localizations of SUMO transcripts is the most important step for increasing our understanding about the mechanisms and functions of SUMOylation. Our current study investigated the functional expression and cellular localization of the new OsSUMO7 proteins in the plants. The DsRed:SUMO7 fusion proteins were localized mainly in nucleus, and formed nuclear subdomain structures (Figure 6A). These nuclear subdomains were observed in a previous study [15]. Our results demonstrated that DsRed-fused OsSUMO7 was processed, activated, and bound to certain substrate proteins by the SUMO-activating system inside cells. Deletion of the GG motif suppressed the accumulation of the *DsRed:SUMO7*Δ*GG* proteins in the nucleus and caused the subnuclear structures to disappear (Figure 6B). These results indicated that the GG motif is necessary for the processing, activation, and accumulation of SUMO proteins in the nucleus. Similar results have been reported previously [23,37]. Additionally, our results confirmed that the OsSUMO7 proteins did not localize at any other organellar cell, except the nucleus (Figure 8). Previous studies found that the fusion proteins GFP:OsSUMO1/2/3 were mainly localized in the nucleus of rice root cells compared with the negative control GFP protein, which was detected in all cell areas, while the fusion protein GFP:OsSUMO4 was found to be localized in specific compartments of the nucleus compared with the GFP protein alone, which was distributed evenly in areas of the cell [50]. This result revealed the essential role of SUMO proteins in the regulation of protein SUMOylation, interaction, and cellular localization of the proteins.

SUMO-conjugating enzyme GFP:SCE1a and DsRed:SUMO7 co-expressing analyses showed that both signals are co-localized in the nucleus and formed nuclear subdomains (Figure 8). This suggested that the OsSUMO7 precursor is processed, activated, and transported to the nucleus through the onion cell SUMOylation system, and that OsSUMO7 possibly binds to the substrate proteins of onion cells. In the rice genome, three conjugation enzyme-encoding genes *OsSCE1, OsSCE2,* and *OsSCE3* were identified [52]. The cellular localization of OsSCE1:GFP fusion proteins was detected in specific compartments in the nucleus, while the fusion proteins of OsSCE2:GFP and OsSCE3:GFP were distributed evenly in the nucleus [52]. The same study suggested that *OsSCE1* is involved in carbohydrate metabolism, *OsSCE3* is associated with drought stress response, while *OsSCE2* still has an unknown function. Three genes of *OsSCE1*, namely *OsSCE1a*, *OsSCE1b*, and *OsSCE1c*, were assessed from rice [22]. SCE1 has a potential role in mediating the conjugation of SUMO to target proteins during the SUMOylation pathway [53]. The accumulations of SUMO conjugates are involved in abiotic stress responses in plants [52,53,54,55]. The modifications of SUMO isoforms are able to regulate the proteins’ activities, interactions among proteins, and the intracellular localization of the proteins [6,50]. Humans and plants have multi-SUMO isoforms, in contrast with yeast which has a single form of a SUMO-encoding gene [6,56]. In humans, the difference in function among SUMO isoforms might be related to differences in their expression levels [5]. The rice genome includes diversification of SUMO genes. However, the SUMO isoforms’ function is still unclear in rice.

## 5. Conclusions

In conclusion, the new *OsSUMO7* gene was defined in the rice genome and its putative protein was characterized. *OsSUMO7* was expressed in the whole plant body. The cellular localization of its expression proteins was mainly localized in the nucleus of the plant cell. The fused proteins of GFP:SCE1a and DsRed:SUMO7 were co-expressed and co-localized in the nucleus, and formed nuclear subdomains. This finding demonstrated that the new OsSUMO7 precursor is able to be processed, activated, and transported to the nucleus through the plant cell SUMOylation system.

## Figures and Tables

**Table 1:** Putative protein characterization of identified rice SUMO genes.

OsSUMO	GenBank	No. of AA	Chr. No.	SUMOplot^TM^ Prediction of SUMOylation Sites					
				Motifs with High Probability			Motifs with Low Probability		
				Motif	Pos.	Score	Motif	Pos.	Score
OsSUMO1	P55857	100	1				**DKKP**	K9	0.39
OsSUMO2	Q8RZU9	101	1				**EKKP**	K10	0.39
OsSUMO3	Q6ZL08	110	7	**VKVE**	K12	0.93	**GKTP**	K20	0.57
OsSUMO4	BAB84389.1	114	1	**VKVE**	K22	0.93			
OsSUMO5	XP_015646034.1	110	7	**LKGD and AKAP**	K72 and K53	0.91 and 0.69			
OsSUMO6	XP_025882647.1	130	7	**VKVE**	K12	0.93	**WKTP and WKAP**	K20 and K28	0.54 and 0.54
OsSUMO7	Query	100	7	**LKGD**	K58	0.91	**HKVD**	K23	0.52

**Table 2:** Physiochemical Properties of OsSUMOs and AtSUMOs by ProtParam.

	Formula	MW (kDa)	T. Pi	TNNCR	TNPCR	Ii	Stability	Ai	GRAVY
OsSUMO1	C_464_H_744_N_138_O_153_S_7_	10.93	4.95	18	13	51.75	Unstable	64.5	−0.681
OsSUMO2	C_471_H_756_N_138_O_156_S_5_	11.01	5.1	17	13	54.05	Unstable	66.73	−0.721
OsSUMO3	C_542_H_834_N_156_O_173_S_4_	12.43	4.77	21	15	42.58	Unstable	59.45	−0.718
OsSUMO4	C_532_H_859_N_145_O_176_S_5_	12.26	4.73	17	12	37.37	Stable	80.44	−0.264
OsSUMO5	C_545_H_858_N_142_O_164_S_10_	12.34	5.01	16	12	37.87	Stable	71.73	−0.174
OsSUMO6	C_657_H_995_N_183_O_195_S_4_	14.71	5.23	19	16	41.52	Unstable	62.31	−0.605
OsSUMO7	C_492_H_795_N_127_O_146_S_5_	10.99	4.92	17	11	30.35	Stable	111.9	0.031
AtSUMO1	C_462_H_740_N_140_O_158_S_6_	10.98	4.91	18	13	47.48	Unstable	59.60	−0.84
AtSUMO2	C_578_H_903_N_159_O_177_S_6_	13.10	5.13	19	15	31.96	Stable	72.33	−0.47
AtSUMO3	C_550_H_866_N_154_O_170_S_7_	12.58	5.09	19	15	47.57	Unstable	76.49	−0.58
AtSUMO4	C_586_H_939_N_173_O_185_S_5_	13.53	6.85	21	21	49.47	Unstable	65.73	−0.85
AtSUMO5	C_524_H_844_N_148_O_164_S_8_	12.10	8.91	12	15	51.41	Unstable	64.07	−0.61
AtSUMO6	C_588_H_934_N_170_O_175_S_7_	13.41	9.13	18	21	43.92	Unstable	62.28	−0.76
AtSUMO7	C_478_H_756_N_136_O_145_S_6_	10.92	6.03	15	13	30.69	Stable	76.00	−0.55
AtSUMO8	C_498_H_782_N_138_O_150_S_5_	11.26	6.05	16	14	53.29	Unstable	76.29	−0.56

MW, molecular weight; T. Pi, theoretical pI; TNNCR, total number of negatively charged residues (Asp + Glu); TNPCR, total number of positively charged residues (Arg + Lys); Ii, instability index; Ai, aliphatic index; and GRAVY, grand average of hydropathicity.

## Data Availability

All data, tables, and figures are original.

## Supplementary Materials

The following supporting information can be downloaded at: https://www.mdpi.com/article/10.3390/biology11010053/s1. Table S1. Primer list used in the study. Table S2. OsSUMO and AtSUMO amino acid and atomic composition. Table S3. OsSUMO and AtSUMO composition of amino acid by percentage. Figure S1: (A) Construction of expression plasmid harboring *OsSUMO7* gene. (B) Construction of expression plasmid harboring *OsSUMO7* gene with GG deletion. (C) Construction of expression plasmid harboring SCE1a gene. (D) Construction of expression plasmid PTS2:GFP. (E) Construction of expression plasmid mt:GFP. (F) Construction of expression plasmid WxTP:GFP. (G) Construction of expression plasmid SYP31:GFP.
