# Supplementary material for: Molecular Characterization and Functional Localization of a Novel SUMOylation Gene in Oryza sativa
Source: Biology (Basel). 2021 Dec 31;11(1):53. doi: 10.3390/biology11010053 (PMC8772976; doi:10.3390/biology11010053)
Supplement: Supplementary file 1 [file biology-11-00053-s001.zip › biology-1492431-supplementary.pdf]

Table S1. Primer list used in the study.

primer name	primer sequence
DsRed T-de F	5' GTTCCTGAGCGGCCGCATGT 3'
DsRed T-de R	5' GGCCGCTCAGGAACAGGTGG 3'
cDNA DsRed T-de F	5' GTTCCTGTAGCGGCCGCATGT 3'
cDNA DsRed T-de R	5' GGCCGCTACAGGAACAGGTGG 3'
OsSUMO7 F	5' TGCCTTCCATCGTTGTGTTG '3
OsSUMO7 R	5' CCATATCAACTTCAGCAGATTCC '3
RT- OsSUMO7 F	5′ GGGTTGATTGATGGTGGTGG ′3
RT- OsSUMO7 R	5′ GTGAAACGGAGGAAGTAGCTC ′3

(OsSUMO7)

Table S2. OsSUMO & AtSUMO Amino Acid and Atomic composition.

	AC					TNA	AAC																				NAA
	C*	H*	N*	O*	S*		A	R	N	D	C	Q	E	G	H	I	L	K	M	F	P	S	T	W	Y	V	
**OsSUMO1**	464	744	138	153	7	1506	9	6	4	9	2	5	9	13	2	4	8	7	5	4	3	3	3	0	1	3	100
**OsSUMO2**	471	756	138	156	5	1526	8	5	4	9	1	6	8	12	2	5	8	8	4	4	4	6	3	0	1	3	101
**OsSUMO3**	542	834	156	173	4	1709	10	11	0	11	0	5	10	14	1	2	7	4	4	5	2	2	8	2	5	7	110
**OsSUMO4**	532	859	145	176	5	1717	10	5	1	11	3	6	6	9	1	7	8	7	2	4	6	7	11	0	2	8	114
**OsSUMO5**	545	858	142	164	10	1719	5	6	1	11	1	3	5	10	2	6	7	6	9	7	6	4	10	0	3	8	110
**OsSUMO6**	657	995	183	195	4	2034	11	11	2	7	0	5	12	16	1	3	9	5	4	5	6	5	9	6	5	8	130
**OsSUMO7**	492	795	127	146	5	1565	2	2	3	8	2	1	9	12	3	3	17	9	3	3	3	4	3	2	0	11	100
**AtSUMO1**	462	740	140	158	6	1506	8	6	5	11	1	6	7	12	2	4	7	7	5	4	2	5	4	0	1	3	100
**AtSUMO2**	578	903	159	177	6	1823	8	6	5	11	2	6	8	10	2	5	10	9	4	9	3	4	6	1	1	6	116
**AtSUMO3**	550	866	154	170	7	1747	8	6	5	13	2	6	6	9	2	5	11	9	5	5	4	5	1	1	3	5	111
**AtSUMO4**	586	939	173	185	5	1888	4	11	3	9	0	3	12	9	3	4	8	10	5	5	2	10	6	1	3	9	117
**AtSUMO5**	524	844	148	164	8	1688	6	5	3	4	2	4	8	7	4	4	7	10	6	2	5	11	8	0	5	7	108
**AtSUMO6**	588	934	170	175	7	1874	2	11	3	8	1	4	10	8	2	4	7	10	6	7	3	8	7	2	2	9	114
**AtSUMO7**	478	756	136	145	6	1521	6	6	2	11	2	6	4	4	4	10	4	7	4	5	5	6	3	0	2	4	95
**AtSUMO8**	498	782	138	150	5	1573	2	6	4	8	1	3	8	6	4	6	8	8	4	7	3	8	3	0	2	6	97

AC Atomic composition; C* Carbon; H* Hydrogen; N* Nitrogen; O* Oxygen; S* Sulfur; TNA Total number of atoms; AAC Amino acid composition; A Alanine; R Arginine; N Asparagin; D Aspartic; C Cysteine; Q Glutamin; E Glutamic; G Glycine; H Histidine; I Isoleucine; L Leucine; K Lysine; M Methionine; F Phenylalanine; P Proline; S Serine; T Threonine; W Tryptophan; Y Tyrosin; V Valine; NAA Number of amino acids.

A Alanine; R Arginine; N Asparagin; D Aspartic; C Cysteine; Q Glutamin; E Glutamic; G Glycine; H Histidine; I Isoleucine; L Leucine; K Lysine; M Methionine; F Phenylalanine; P Proline; S Serine; T Threonine; W Tryptophan; Y Tyrosin; V Valin

(OsSUMO7)

Table S3. OsSUMO and AtSUMO composition of amino acid by percentage.

	A	R	N	D	C	Q	E	G	H	I	L	K	M	F	P	S	T	W	Y	V
**OsSUMO1**	9.00%	6.00%	4.00%	9.00%	2.00%	5.00%	9.00%	13.00%	2.00%	4.00%	8.00%	7.00%	5.00%	4.00%	3.00%	3.00%	3.00%	0.00%	1.00%	3.00%
**OsSUMO2**	7.90%	5.00%	4.00%	8.90%	1.00%	5.90%	7.90%	11.90%	2.00%	5.00%	7.90%	7.90%	4.00%	4.00%	4.00%	5.90%	3.00%	0.00%	1.00%	3.00%
**OsSUMO3**	9.10%	10.00%	0.00%	10.00%	0.00%	4.50%	9.10%	12.70%	0.90%	1.80%	6.40%	3.60%	3.60%	4.50%	1.80%	1.80%	7.30%	1.80%	4.50%	6.40%
**OsSUMO4**	8.80%	4.40%	0.90%	9.60%	2.60%	5.30%	5.30%	7.90%	0.90%	6.10%	7.00%	6.10%	1.80%	3.50%	5.30%	6.10%	9.60%	0.00%	1.80%	7.00%
**OsSUMO5**	4.50%	5.50%	0.90%	10.00%	0.90%	2.70%	4.50%	9.10%	1.80%	5.50%	6.40%	5.50%	8.20%	6.40%	5.50%	3.60%	9.10%	0.00%	2.70%	7.30%
**OsSUMO6**	8.50%	8.50%	1.50%	5.40%	0.00%	3.80%	9.20%	12.30%	0.80%	2.30%	6.90%	3.80%	3.10%	3.80%	4.60%	3.80%	6.90%	4.60%	3.80%	6.20%
**OsSUMO7**	2.00%	2.00%	3.00%	8.00%	2.00%	1.00%	9.00%	12.00%	3.00%	3.00%	17.00%	9.00%	3.00%	3.00%	3.00%	4.00%	3.00%	2.00%	0.00%	11.00%
**AtSUMO1**	8.00%	6.00%	5.00%	11.00%	1.00%	6.00%	7.00%	12.00%	2.00%	4.00%	7.00%	7.00%	5.00%	4.00%	2.00%	5.00%	4.00%	0.00%	1.00%	3.00%
**AtSUMO2**	6.90%	5.20%	4.30%	9.50%	1.70%	5.20%	6.90%	8.60%	1.70%	4.30%	8.60%	7.80%	3.40%	7.80%	2.60%	3.40%	5.20%	0.90%	0.90%	5.20%
**AtSUMO3**	7.20%	5.40%	4.50%	11.70%	1.80%	5.40%	5.40%	8.10%	1.80%	4.50%	9.90%	8.10%	4.50%	4.50%	3.60%	4.50%	0.90%	0.90%	2.70%	4.50%
**AtSUMO4**	3.40%	9.40%	2.60%	7.70%	0.00%	2.60%	10.30%	7.70%	2.60%	3.40%	6.80%	8.50%	4.30%	4.30%	1.70%	8.50%	5.10%	0.90%	2.60%	7.70%
**AtSUMO5**	5.60%	4.60%	2.80%	3.70%	1.90%	3.70%	7.40%	6.50%	3.70%	3.70%	6.50%	9.30%	5.60%	1.90%	4.60%	10.20%	7.40%	0.00%	4.60%	6.50%
**AtSUMO6**	1.80%	9.60%	2.60%	7.00%	0.90%	3.50%	8.80%	7.00%	1.80%	3.50%	6.10%	8.80%	5.30%	6.10%	2.60%	7.00%	6.10%	1.80%	1.80%	7.90%
**AtSUMO7**	6.30%	6.30%	2.10%	11.60%	2.10%	6.30%	4.20%	4.20%	4.20%	10.50%	4.20%	7.40%	4.20%	5.30%	5.30%	6.30%	3.20%	0.00%	2.10%	4.20%
**AtSUMO8**	2.10%	6.20%	4.10%	8.20%	1.00%	3.10%	8.20%	6.20%	4.10%	6.20%	8.20%	8.20%	4.10%	7.20%	3.10%	8.20%	3.10%	0.00%	2.10%	6.20%

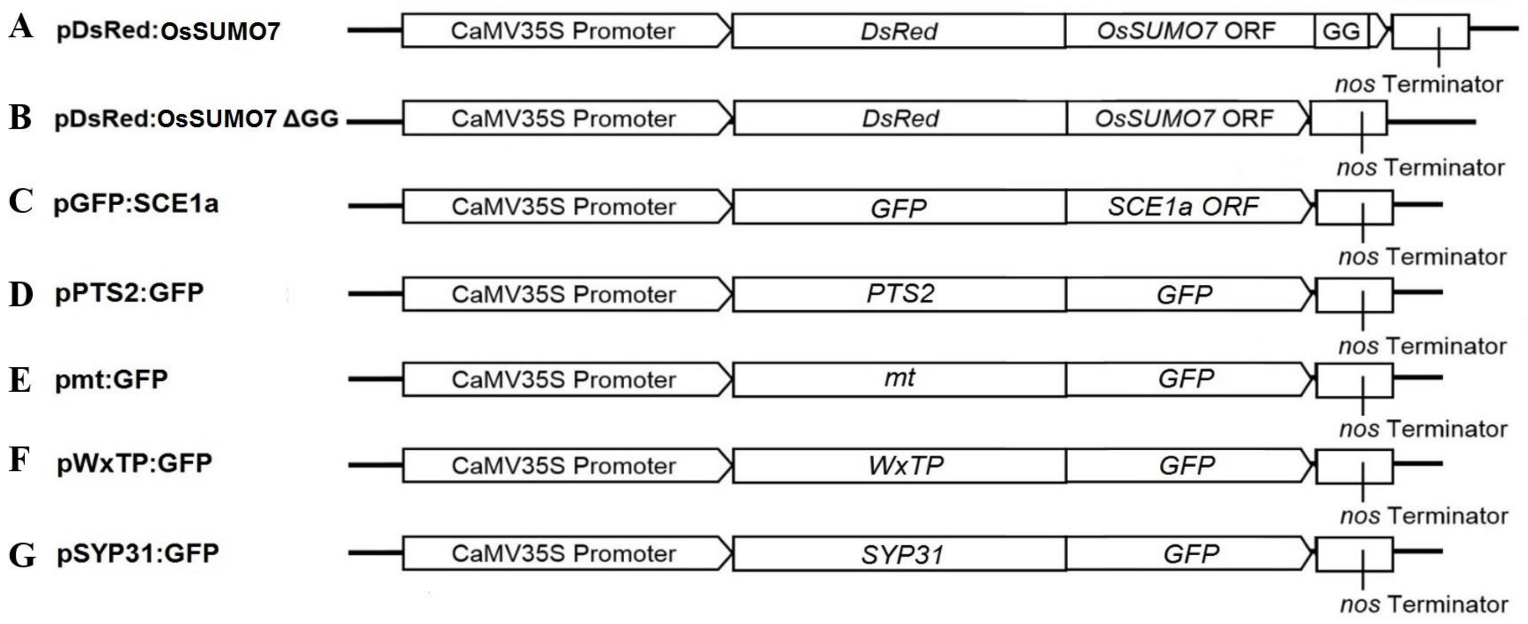

Figure S1. A) Construction of expression plasmid harboring OsSUMO7 gene. B) Construction of expression plasmid harboring OsSUMO7 gene with GG Deletion. C) Construction of expression plasmid harboring SCE1a gene. D) Construction of expression plasmid PTS2:GFP. E) Construction of expression plasmid mt:GFP. F) Construction of expression plasmid WxTP:GFP. G) Construction of expression plasmid SYP31:GFP.
